# Stakeholder perspectives on job analysis for disability-inclusive sewing employment in China: a qualitative content analysis

**DOI:** 10.3389/fresc.2026.1725150

**Published:** 2026-07-09

**Authors:** Huiling Hu, Ziyu Wan, Peter H. F. Ng, Karen P. Y. Liu, Eugene Yujun Fu, Andy S. K. Cheng

**Affiliations:** 1 Department of Rehabilitation Sciences, The Hong Kong Polytechnic University, Hong Kong, Hong Kong SAR, China; 2 Department of Computing, The Hong Kong Polytechnic University, Hong Kong, Hong Kong SAR, China; 3 Centre for Learning, Teaching and Technology (LTTC), The Education University of Hong Kong, Hong Kong, Hong Kong SAR, China; 4 Department of Health and Physical Education, The Education University of Hong Kong, Hong Kong, Hong Kong SAR, China

**Keywords:** employment, job analysis, sewing, vocational evaluation, work ability

## Abstract

**Introduction:**

This study explored stakeholder-informed perspectives on sewing employment in China, with a focus on employment-related factors, critical work tasks, and job demand domains. The findings were intended to inform future vocational evaluation and the development of AI-enhanced job analysis approaches for people with disabilities.

**Methods:**

An exploratory qualitative study using qualitative content analysis was conducted. Data were collected through three dyadic stakeholder interviews, each involving one sewing worker and one manager, and four one-on-one semi-structured interviews with vocational rehabilitation professionals.

**Results:**

The analysis identified fourteen employment-related categories, four critical work tasks, and four job demand domains relevant to sewing work.

**Conclusion:**

This study identified employment-related categories, critical work tasks, and job demand domains in disability-inclusive sewing employment in China. These findings may inform future vocational evaluation and AI-enhanced job analysis, with video-based analysis positioned as a complementary tool for quantifying observable physical job demands.

## 1 Introduction

Work is a vital occupation that supports individual well-being, family life, and social participation (1, 2). For people with disabilities (PWD), employment is associated with improved health, economic independence, and social inclusion, yet they continue to face barriers to obtaining and sustaining suitable work (3, 4). In China, disability employment has become an important policy and service priority. Although national policies have strengthened employment support for PWD, previous research in mainland China suggests that policy support does not always translate into positive employment outcomes, partly because of persistent challenges in policy implementation, employer engagement, workplace support, and person–job matching (5). National data indicate that China had nearly 18 million working-age persons with certified disabilities (6). By the end of 2021, 8.816 million were employed in urban and rural areas, while more than eight million had not achieved employment (6). More recently, the 2025 Statistical Bulletin on the Development of Undertakings for Persons with Disabilities in China reported that 448,000 persons with certified disabilities newly obtained employment in 2025, and the total number of employed persons with certified disabilities reached 8.915 million nationwide (7). While these figures reflect important progress, they also highlight the continuing need to improve the precision and quality of employment support. A persistent challenge is that employment support programs often focus on increasing employment opportunities, while less attention is given to systematically understanding job demands and matching these demands with the abilities of individual job seekers. Recent national policy has therefore emphasized more individualized and refined employment services, including “one person, one plan” support, vocational capacity improvement, and the development of vocational ability assessment systems (8). These policy priorities underscore the need for context-sensitive and evidence-based approaches to job analysis, vocational evaluation, and person–job matching, particularly for PWD whose employment outcomes may be strongly influenced by the interaction between individual abilities, workplace environments, and job requirements.

Global frameworks have long promoted inclusive employment for PWD, as reflected in the Sustainable Development Goals (SDGs), particularly SDG 8 (decent work) and SDG 10 (reduced inequalities), which reinforce the global commitment to “leave no one behind” (9). International Labor Organization Convention No. 159 and the Convention on the Rights of PWD Article 27 affirm the right to work and advocate for accessible vocational rehabilitation and equitable labor policies (10, 11). Vocational evaluation plays a central role in vocational rehabilitation by assessing how an individual's physical, cognitive, interpersonal, and emotional abilities align with job demands (12, 13). This process allows vocational rehabilitation professionals to provide individualized career guidance (14). Job analysis is a systematic process used in vocational evaluation to identify job demands (15). This process involves identifying the duties, responsibilities, and competencies required for the intended job position and any potential challenges and risks under the work conditions (16, 17). However, job analysis based primarily on interviews and unstructured observation may be affected by subjectivity, inconsistency, and limited precision (18–20), particularly when assessors need to estimate fine-grained task demands or when standardized and locally relevant occupational information is unavailable (21, 22).

Our broader research project explores the development of the Smart Evaluation of Work Ability (SEWAbility) system, which uses artificial intelligence (AI), including computer vision and machine learning, to support the analysis of observable job tasks based on real-world video data (23). As a preliminary step in system design, we selected sewing work as an exploratory case because of its relevance in China's labor market, where sewing machine operators have been ranked 16th among the top 100 most in-demand jobs (24), and because its relatively stable indoor environment and repetitive task structure make it suitable for video-based observation (25). Despite high demand, sewing work in China lacks context-specific and well-documented job analysis that can inform vocational evaluation for PWD. To address this gap, this study explored stakeholder-informed perspectives on sewing employment, critical work tasks, and job demand domains through dyadic stakeholder interviews with sewing workers and managers and individual interviews with vocational rehabilitation professionals. This exploratory qualitative content analysis aimed to generate context-specific insights that may inform future vocational evaluation and AI-enhanced job analysis research.

Specifically, this study was designed to explore the following research questions (RQs):

RQ1: What factors influence the employment experience of sewing workers with disabilities?
RQ2: What are the critical work tasks involved in sewing work?
RQ3: What job demand domains are required for performing sewing work from a vocational evaluation perspective?

## 2 Methods

This study was conducted in August and September 2024 in a central inland region of China with a strong garment manufacturing presence. A two-tiered data collection strategy was adopted. The first tier consisted of three dyadic stakeholder interviews, each involving one sewing worker and one manager, to explore employment-related factors and critical work tasks (RQ1 and RQ2). The second tier comprised four semi-structured individual interviews with vocational rehabilitation professionals, focusing on job demand domains associated with sewing work (RQ3).

### 2.1 Context and research team

This study is part of a broader research project on developing an AI-enhanced vocational evaluation system to support the objectivity and scalability of vocational evaluation for PWD. Findings from this study are intended to inform future system design, particularly by identifying context-specific employment-related categories, critical work tasks, and job demand domains relevant to sewing work.

To construct shared understanding (26), our interdisciplinary research team held regular meetings and jointly developed the semi-structured interview guide. The team included two PhD students in rehabilitation sciences (HH, ZW), two computer science assistant professors (YF, PN), and two occupational therapy full professors (KL, AC), comprising five male (HH, ZW, PN, YF, AC) and one female researcher (KL). We also conducted simulated qualitative interview sessions to refine the question design, anticipate potential challenges, and align our analytical approach. These collaborative strategies supported methodological coherence and contributed to the practical relevance of the study within the broader PhD research framework.

### 2.2 Study design

This study employed an exploratory qualitative design using purposive sampling to recruit participants for dyadic stakeholder interviews and individual interviews. RQ1 explored factors influencing the employment experience of sewing workers with disabilities, while RQ2 focused on identifying critical work tasks involved in sewing work. Sewing workers and managers were invited to participate in dyadic stakeholder interviews because they were key workplace stakeholders with direct experience of sewing production, daily supervision, training, and performance evaluation. Dyadic stakeholder interviews allow two participants with relevant shared workplace experience to interact and elaborate on their perspectives within the same session (27). This format enabled the study to capture how different workplace stakeholders described sewing employment and work tasks. RQ3 addressed job demand domains from a vocational evaluation perspective. Given the domain-specific expertise of vocational rehabilitation professionals and the potential variation among assessors, one-on-one interviews were used to allow independent, in-depth responses.

Data were analyzed using qualitative content analysis (28), and the study followed the Consolidated Criteria for Reporting Qualitative Research (COREQ) (29). Qualitative content analysis was selected because it enables the systematic classification of interview data into categories and domains that represent similar meanings (28). Following Hsieh and Shannon's typology of qualitative content analysis, a combined directed and inductive approach was adopted (28).

RQ1 was analyzed using directed qualitative content analysis informed by the Person–Environment–Occupation–Performance (PEOP) model. PEOP was selected because RQ1 sought to identify and organize employment-related factors within the Person, Environment, and Occupation domains, with employment performance conceptualized as the outcome of interactions among these domains. Both PEOP and the International Classification of Functioning, Disability and Health (ICF) recognize that functioning and participation are influenced by interactions between individual and contextual factors (30–32). However, whereas the ICF provides a standardized framework and terminology for describing functioning, disability, and contextual factors (32), PEOP offers an occupation-focused structure that was more directly aligned with the analytic purpose of RQ1. Accordingly, PEOP, rather than the ICF, was used to structure the coding process. Performance referred to the ability to obtain and sustain sewing employment and was treated as the outcome of interactions among the Person, Environment, and Occupation domains rather than as a separate coding category. RQ2 and RQ3 were analyzed using inductive qualitative content analysis.

### 2.3 Recruitment, participants and study setting

This study was conducted in collaboration with a provincial non-profit organization in China that supports PWD. As a key promoter of rehabilitation and employment-related services across the province, the organization facilitated access to suitable study sites and participants. To ensure relevance to the study aim, only textile enterprises employing PWD were considered. Purposive sampling was used to identify an information-rich workplace case relevant to disability-inclusive sewing employment. The organization also introduced the research team to participants in advance to build rapport and ensure informed consent.

The participating site was a disability-inclusive commercial home-textile production enterprise in China. The company has been recognized as a “National Base for Proportional Employment of Persons with Disabilities” by the Employment Service Guidance Center of the China Disabled Persons' Federation (33). Although the enterprise operated as a commercial production workplace, it was not representative of a typical sewing factory in terms of its workforce composition and support arrangements. More than 70% of its employees were persons with disabilities, and the company had cumulatively provided employment opportunities for more than 470 persons with disabilities and veterans (34). To support an inclusive work environment, the workplace had implemented accessibility-related modifications, including elevators, air conditioning in production and living areas, accessible facilities, and dedicated spaces for staff support, training, counseling, recreation, laundry, fitness, and accommodation. Although the company separately operated an auxiliary employment service center, the present study focused exclusively on its commercial sewing production workplace; the auxiliary employment service center was outside the scope of this study.

Participants in the dyadic stakeholder interviews included three sewing workers (W1–W3) and three managers (M1–M3). Workers were eligible if they had more than one year of employment experience in the factory, which was considered relevant for discussing work stability and employment experience. Managers were selected based on their familiarity with sewing tasks, worker training, daily supervision, and performance evaluation. The dyadic stakeholder interviews were designed to explore employment-related factors and critical sewing work tasks from workplace stakeholders with direct experience of sewing production and management. Among the worker and manager participants, two female participants (W3 and M3) reported holding CDPF disability certificates for Grade 4 physical disabilities. Under the CDPF classification system, disability severity ranges from Grade 1 (most severe) to Grade 4 (least severe) (35). Participants in the one-on-one interviews included four vocational rehabilitation professionals (V1–V4) recruited from a rehabilitation hospital and a provincial employment service center for PWD. All had at least five years of field experience. They had an average of 9.5 years of professional experience. A summary of participant characteristics is provided in Table 1. All participants provided informed consent, and none withdrew from the study.

**Table 1:** Characteristics of included participants.

ID	Gender	Age	Years of experience	Self-reported disability status	Self-reported CDPF disability certificate status	Disability type and CDPF grade
W1	Male	31	6	Reported no disability	No	N/A
W2	Male	35	8	Reported no disability	No	N/A
W3	Female	41	10	Reported disability	Yes	Physical disability, grade 4
M1	Male	38	10	Reported no disability	No	N/A
M2	Male	46	11	Reported no disability	No	N/A
M3	Female	51	21	Reported disability	Yes	Physical disability, grade 4
V1	Male	31	5	N/A	N/A	N/A
V2	Male	33	5	N/A	N/A	N/A
V3	Female	36	7	N/A	N/A	N/A
V4	Female	48	21	N/A	N/A	N/A

CDPF, China Disabled Persons' Federation; N/A, not applicable. In the CDPF disability classification system, disability severity is categorized from Grade 1 (most severe) to Grade 4 (least severe).

### 2.4 Procedure

The study was approved by the Department of Rehabilitation Sciences Ethics Committee at The Hong Kong Polytechnic University (Ethics Approval Code: HSEARS20240815005). All study procedures were conducted in accordance with relevant guidelines and regulations and adhered to the Declaration of Helsinki. A semi-structured interview guide was developed to ensure consistency with the research aims while allowing participants to elaborate on their experiences and perspectives. The interview topics are shown in Table 2.

**Table 2:** Interview topics for the dyadic stakeholder interviews and individual interviews.

Dyadic stakeholder interviews with sewing workers and managers	Interview with vocational rehabilitation professionals
1. For people with disabilities, what do you think can affect their career as a sewing worker?	1. Based on the work routines of sewing workers, what abilities do you think are necessary for their successful employment?
2. Based on your experience, what are the primary personal factors that influence the successful employment of sewing workers?	2. Based on these critical work tasks, what job demands do you think are required?
3. Are there environmental factors critical to the successful employment of sewing workers?	3. Have you observed any other abilities or characteristics crucial to this work?
4. In your opinion, please briefly describe the types of activities you would say are essential to working as a sewing worker.	4. Based on your professional experience, how would you evaluate a client's work ability if their intended occupation were sewing work?
5. What are the basic tasks and critical work tasks for a sewing worker in your factory?	5. What convinces you that one person is appropriate to work as a sewing worker?
6. What additional thoughts do you want to mention that are important for employment as a sewing worker?	6. What additional suggestions do you have for job analysis for a sewing worker?

Three dyadic stakeholder interviews were held in a meeting room at the textile corporation. Each dyadic interview involved one sewing worker and one manager. The moderator (HH), a trained health sciences researcher, had visited the site before data collection and was familiar with the workplace context, which helped facilitate rapport and contextual understanding during the interviews. Open-ended questions were used to explore employment-related factors and critical work tasks related to sewing work (RQ1 and RQ2). Four individual interviews with vocational rehabilitation professionals were conducted in a rehabilitation hospital and an employment service center. During these four interviews, the moderator shared workplace videos and preliminary findings from the dyadic stakeholder interviews to stimulate discussion of job demand domains associated with sewing work (RQ3). The use of workplace videos was intended to provide a common reference point for discussing observable work tasks and job demands. Each interview session lasted approximately 80–100 min.

### 2.5 Data collection and analysis

All sessions were audio-recorded with participants' consent and supplemented by detailed field notes. Each session was facilitated by a trained moderator (HH) and supported by a note-taker (ZW), who recorded written observations to complement the audio recordings. Audio recordings from the dyadic stakeholder interviews and individual interviews were transcribed verbatim by two researchers (HH, ZW), both trained in healthcare and qualitative research. Transcripts were read repeatedly to support familiarization with the data. HH and ZW documented analytic memos and initial reflections throughout the analysis process.

Coding and data management were supported using NVivo Release 15.0.0. HH and ZW independently conducted line-by-line coding. For RQ1, initial coding was guided by the PEOP framework, with codes first organized under the Person, Environment, and Occupation domains. Codes with unclear or overlapping domain assignments were discussed and resolved through team review. For RQ2 and RQ3, codes were generated inductively from participants' accounts and then grouped into broader categories or domains. HH grouped the initial codes into candidate categories, which were collaboratively reviewed by ZW and a trained research assistant. During analysis, the research team monitored whether new codes or categories continued to emerge from the available data. Given the small and context-specific sample, we did not claim broad theoretical saturation. Instead, data adequacy was considered in relation to the focused exploratory aims, the participants' direct knowledge of sewing work or vocational rehabilitation, and the relevance of the available data to the research questions (36). Within the available dataset, the final dyadic stakeholder interview and the final vocational rehabilitation professional interview did not generate substantially new codes or categories relevant to the research questions. This was interpreted as sufficient data adequacy for the exploratory aims of the study, rather than as saturation across wider populations or workplace settings.

The candidate categories were then reviewed for internal coherence, distinctiveness, and relevance to the research questions. HH and ZW refined the category structure, with additional feedback from YF to enhance conceptual clarity and practical relevance. Category labels and descriptions were finalized to reflect their meanings in relation to the study aims. Senior team members (PN, KL, AC) provided further feedback to refine the conceptual clarity and practical applicability of the findings. HH drafted the Results section with support from ZW, integrating illustrative participant quotations for each category or domain. The draft underwent technical and conceptual review by PN and AC.

Examples of the coding process and category development are presented in Table 3.

**Table 3:** Examples of codes and category development.

Quotations	Code	Category
“…some people just want a paycheck… they might leave once they find an easier job with similar pay, like a front desk role. But those who enjoy sewing take pride in improving their skills, and that shows in the quality of their work and their willingness to stay and grow with us…” (M1)	Motivation rooted in personal interest and pride contributes to better work performance.	Work willingness and motivation
“… workers with visual impairments struggle with aligning stitches accurately… but those who've lost part of their fingers can still operate the sewing machines quite well… it really depends on what kind of impairment they have and how it affects the task….” (M2)	Type of impairment determines specific task compatibility	Disability type and severity

### 2.6 Rigor and trustworthiness

To ensure research rigor, we adhered to established criteria for qualitative trustworthiness, including credibility, dependability, transferability, and confirmability (37, 38). This two-tiered study followed a consistent semi-structured interview guide developed collaboratively by an interdisciplinary team, ensuring alignment with the research objectives and comparability across sessions. All interviews were conducted in Chinese, audio-recorded with consent, and transcribed verbatim. To support credibility during data collection, the moderator conducted real-time participant confirmation at the end of each dyadic stakeholder interview and individual interview. The moderator briefly summarized the key points raised during the session and invited participants to confirm, clarify, or add further information. This procedure helped reduce misunderstandings and supported consistency between the researchers' immediate interpretation and participants' intended meanings. A research assistant verified transcript accuracy. NVivo Release 15.0.0 was used to support systematic coding and data organization. Software-supported coding management was combined with manual verification to enhance coding consistency and traceability. To strengthen credibility, two researchers with qualitative expertise independently reviewed the initial codes and categories. Discrepancies were resolved through iterative team discussions. Preliminary findings were presented at a research seminar attended by 11 interdisciplinary scholars from rehabilitation sciences, occupational therapy, nursing, information technology, organizational psychology, and computer science. Peer debriefing during this session provided constructive feedback, which informed further refinement of the category structure.

Transferability was supported by providing detailed descriptions of the study setting, participant characteristics, sampling criteria, and institutional context of the disability-inclusive factory. These descriptions allow readers to judge the applicability of the findings to other vocational rehabilitation and sewing employment contexts. Dependability was addressed by maintaining a clear audit trail, including records of coding decisions, category refinement, and analytic team discussions. Confirmability was supported through transparent documentation of category development and the use of representative participant quotations. Participant quotations were translated from Chinese to English after the qualitative content analysis was completed to preserve the integrity of the coding process. HH and ZW conducted the translations, which were then reviewed and cross-checked by two senior researchers (YF, PN) to ensure linguistic accuracy and contextual fidelity.

## 3 Results

### 3.1 RQ1: what factors influence the employment experience of sewing workers with disabilities?

The qualitative content analysis identified fourteen employment-related categories, organized under the Person, Environment, and Occupation domains of the PEOP model. Performance was conceptualized as the outcome of these interacting domains, referring to the ability to obtain and sustain sewing employment. The categories are presented in Table 4.

**Table 4:** Employment-related categories affecting sewing employment, organized using the PEOP framework.

Person	Environment	Occupation
Work willingness and motivation	The physical environment of workplace	Occupational safety
Disability type and severity	Labor market environment	Job security
Learning ability	Family attitudes	Mentorship and training program
Personality	Remuneration package	
Motor and sensory ability	Workplace atmosphere	
Professional background		

Performance was conceptualized as the employment outcome shaped by the interaction among Person, Environment, and Occupation factors. In this study, Performance referred to the ability to obtain and sustain sewing employment and was therefore not presented as a separate category.

#### 3.1.1 Work willingness and motivation

Willingness to work and motivation were identified as key Person factors influencing employment. Both managers and sewing workers emphasized that a genuine desire to work played a more decisive role than physical ability alone. “*The worker has to want to work on their own…* *only when they truly want to work can they do a good job*,” *a manager explained, describing motivation as* “*an essential prerequisite*” (M1). A sewing worker shared a similar view: “*I chose to stay not just because I can do the job, but I genuinely want to work as a sewing worker*” (W1). These accounts indicate that participants viewed personal motivation as relevant to job engagement and retention.

#### 3.1.2 Disability type and severity

Disability type and severity were identified as key Person factors influencing suitability for sewing work. Participants noted that both physical and mental impairments affect performance differently, and severity plays a critical role.

“*Sewing takes both mental focus and technical skills…, for some people with mental health conditions, it might not be a good fit, even if they’re physically capable of operating the machine*,” a manager explained (M1). Another manager stated, “*Even those who have lost part of fingers can still work very well..* *that is why we need to consciously consider how the severity will affect the job*” (M2). These accounts indicate that participants perceived work suitability as depending not only on the type of disability but also on how the impairment affected specific sewing tasks and support needs.

#### 3.1.3 Learning ability

Learning ability was identified as a key Person factor influencing employability in sewing work. Both managers and sewing workers described the ability to learn new techniques, adapt to different materials, and respond to changing task requirements as important for maintaining work performance.

A manager explained that sewing requires ongoing adaptation: “*Workers need to learn how to sew different textiles to maintain productivity*” (M3). A sewing worker also acknowledged the value of quick learners, stating, “*Some of our colleagues are quick learners..* *that is great for improving work efficiency*” (W3). These accounts indicate that participants associated learning ability with adaptation to changing materials, task demands, and workplace performance.

#### 3.1.4 Personality

Personality was identified by participants as a Person factor relevant to task performance, learning, and employment retention. Participants described humility, diligence, patience, and willingness to learn from experienced colleagues as helpful for adapting to sewing work.

“*Some workers are just too lazy.. some are too impatient… some are too stubborn, insisting on doing things their way. Their efficiency is low, and the quality of their work suffers*,” a manager noted (M1). A worker stated, “*Newcomers should stay humble and learn from senior colleagues..* *some are too arrogant, which is bad for this work*” (W1). These accounts indicate that participants associated personality and work-related attitudes with collaboration, learning, and job stability.

#### 3.1.5 Motor and sensory ability

Motor and sensory abilities, particularly finger dexterity, tactile perception, and near vision, were identified as key Person factors influencing sewing performance. Participants emphasized the need for precision and coordination.

“*Sewing requires more delicate skills than many other jobs..* *Finger dexterity is significant*,” a manager noted (M1). A worker stated, “*Distinguishing the different fabric textures..* *seeing the needles properly is a basic requirement*” (W1). These accounts indicate that participants viewed fine sensorimotor abilities as relevant to safe and efficient task performance.

#### 3.1.6 Professional background

Professional background was identified as a Person factor affecting employability. Managers described basic textile knowledge or prior sewing experience as helpful for adapting to sewing work.

“*Applicants with basic textile knowledge are preferred, and it's even better if they've worked in sewing before…, they tend to adjust faster*,” a manager explained (M3). Managers described relevant experience as helpful for job readiness and adaptation to sewing work.

#### 3.1.7 Physical environment of workplace

The physical environment and workplace accessibility were identified as Environment factors influencing employment. Both managers and workers described workplace comfort, accessibility, and transportation convenience as supportive of continued work participation.

“*We widened the hallway to accommodate wheelchairs..* *we also have air conditioning, elevators, and washroom grab handles*,” a manager shared (M1). A worker stated, “*Working in a comfortable indoor environment, away from the sun and rain is good..* *also there is a bus stop right at the gate*” (W2). These accounts indicate that participants viewed workplace accessibility, comfort, and transportation convenience as supportive of continued work participation.

#### 3.1.8 Labor market environment

The labor market environment was identified as an important Environment factor influencing sewing worker employment. Both managers and sewing workers noted a labor supply shortage relative to industry demand.

“*There is a shortage of sewing workers in China's labor market*,” a manager stated (M2). A sewing worker recalled (W3), “*Sewing was a common career choice for women*,” indicating that participants also viewed sewing work in relation to changing gendered labor patterns and occupational preferences. These accounts indicate that participants perceived broader labor market trends as relevant to recruitment and employment stability in the sewing industry.

#### 3.1.9 Family attitudes

Family attitudes were identified as an Environment factor influencing employment sustainability. Both managers and sewing workers described family support or interference as relevant to whether individuals remained in sewing work.

A sewing worker shared, “*My family encourages me to work here. They're supportive and happy that I'm doing well*,” emphasizing how family recognition can reinforce job commitment (W3). In contrast, a manager observed, “*Some parents worry that their children are struggling, so they take them home, even when the job is manageable*,” illustrating how overprotection may disrupt employment (M1). These accounts indicate that participants perceived family involvement as potentially supportive or disruptive to sustained work participation.

#### 3.1.10 Remuneration package

Remuneration was identified as a key Environment factor influencing employment decisions. Participants described wages, bonuses, and employee benefits as relevant to attracting and retaining sewing workers.

“*I can earn more here… and we also get bonuses during festivals and on our birthdays*,” a worker shared (W3), highlighting how salary and welfare incentives contributed to job satisfaction. This account indicates that income and welfare benefits were perceived as contributing to job satisfaction and continued employment.

#### 3.1.11 Workplace atmosphere

Workplace atmosphere was identified as an Environment factor influencing employment experience. Sewing workers described an inclusive and supportive interpersonal environment as important to their sense of belonging.

“*The employers and colleagues are easygoing and friendly… no discrimination… we're all the same, like a family*,” one worker shared (W3), highlighting how positive interpersonal dynamics promote a sense of belonging. This account indicates that the worker associated supportive workplace relationships with comfort, belonging, and willingness to remain employed.

#### 3.1.12 Occupational safety

Occupational safety was emphasized as a core Occupation factor influencing employment sustainability. Managers highlighted the inherent risks of sewing work and the need for strict safety measures.

“*Safety comes first… We use needles, scissors, and other sharp tools, so anything that could lead to workplace accidents should be avoided*,” one manager explained (M1), underscoring the importance of hazard prevention in daily operations. This account indicates that participants viewed hazard prevention as important for safe task performance and continued work participation.

#### 3.1.13 Job security

Job security was identified as an Occupation factor influencing employment sustainability. Managers and sewing workers described sewing work in this factory as relatively stable, particularly for older or female workers, compared with more physically demanding jobs.

“*The advantage of this job is its stability since the workload is manageable. Unlike physically demanding jobs, where older female workers might be laid off when they can no longer keep up, this job is more sustainable*,” a worker shared (W3). A manager echoed this view: “*What matters most is sewing efficiency. The longer they work here, the more skilled and faster they become… Yes, we offer stable employment opportunities*” (M1). These accounts indicate that participants perceived sewing work in this factory as relatively stable and sustainable, particularly for workers who may face barriers in more physically demanding employment settings.

#### 3.1.14 Mentorship and training program

Mentorship and training were identified as Occupation factors that support employment and ease the transition for new workers. Both managers and sewing workers described structured guidance from experienced workers as helpful for skill development and workplace adaptation.

“*I knew nothing about sewing… but they assigned a veteran worker to teach me. It helped a lot*,” a worker shared (W3). A manager (M1) stated, “*We provide training and have different product lines… Newcomers can start with simpler tasks under the guidance of experienced workers*”. These accounts indicate that participants viewed mentorship, gradual onboarding, and task progression as supportive of learning, confidence, and retention among new workers.

### 3.2 RQ2: what are the critical work tasks involved in sewing work?

Four critical work tasks were identified: (1) Fabric Side Identification, (2) Manual Stitching Preparation, (3) Electric Sewing Machine Operation, and (4) Instruction-Based Task Execution.

#### 3.2.1 Fabric Side identification

Distinguishing the front and back sides of fabric based on subtle differences in texture, sheen, or pattern was described as a routine but important task in sewing work. Participants noted that accurate identification was necessary for correct assembly and product quality.

“*We often deal with materials that look almost identical on both sides. You need to touch or look closely to figure out which side is right*,” a manager explained (M1).

“*Some fabrics are similar front and back, but once you get used to the feel or shine, you can tell*,” a worker stated (W2).

#### 3.2.2 Manual stitching preparation

Manual preparation tasks such as threading, aligning fabric, and adjusting thread tension were considered fundamental components of the sewing workflow. These tasks directly impact machine performance and product outcomes.

“*You can't just sit at the machine and sew…, need to know how to align the fabric, check the thread, and get the tension right*,” a manager said (M2). “*I always check the thread tension first. If it's off, the stitching will be loose or uneven*,” a worker shared (W3).

#### 3.2.3 Electric sewing machine operation

Operating electric sewing machines was described as a core task in sewing production. Participants noted that successful operation requires rhythm, precision, and familiarity with different product lines.

“*New employees might find the machines fast at first, but once they get used to the rhythm, they can keep up*,” a manager explained (M3).

“*After a while, using the machine becomes automatic. You just keep the speed steady and focus on not making mistakes*,” a worker reflected (W1).

#### 3.2.4 Instruction-based task execution

Executing sewing tasks based on verbal, demonstrated, or written instructions was described as important for maintaining workflow and product quality. Participants also noted that workers sometimes needed to adapt to urgent or changing production requirements.

“*We give them clear steps, sometimes verbally, sometimes with examples. It's important that they follow exactly as told*,” a manager noted (M3).

A worker stated, “*Sometimes they just show me once and I have to remember it… Sometimes the task changes halfway due to urgent orders, so you have to adjust quickly*” (W3).

### 3.3 RQ3: what job demand domains are required for performing sewing work from a vocational evaluation perspective?

Four job demand domains were identified: (1) Gross Motor Ability, (2) Fine Motor Ability, (3) Sensory Ability, and (4) Mental Ability and Communication.

#### 3.3.1 Gross motor ability

Gross motor ability was identified as a physical job demand domain in sewing work. Vocational rehabilitation professionals described sewing tasks as requiring prolonged sitting, postural control, upper-limb movement for pushing fabric, bending to pick up materials, and rhythmic coordination when operating foot pedals.

“*A sewing worker needs whole-body coordination…, pushing fabric, bending over to collect materials, and managing the foot pedal simultaneously…*” a vocational rehabilitation professional explained (V1).

Another stated, “*They must maintain posture for long hours while continuously moving their upper and lower limbs. This poses challenges for individuals with motor impairments*” (V2).

#### 3.3.2 Fine motor ability

Fine motor ability was identified as a job demand domain related to accurate manipulation of fabric, tools, and machine components. Tasks such as reaching, grasping, handling, threading, positioning, and adjusting materials require dexterity, precision, and hand-eye coordination.

“*Precise hand movements are needed to thread needles, position fabric, and manage fine adjustments during sewing*,” one expert noted (V1).

#### 3.3.3 Sensory ability

Sensory ability was identified as a job demand domain relevant to sewing accuracy and material alignment. Vocational rehabilitation professionals described near vision, depth perception, color discrimination, and tactile perception as important for identifying fabric characteristics, aligning seams, and detecting errors.

“*Sewing tasks rely heavily on visual discrimination and tactile feedback, for example, distinguishing fabric sides by touch or aligning seams visually*,” one expert stated (V3).

#### 3.3.4 Mental ability and communication

Mental ability and communication were identified as job demand domains in sewing work. Vocational rehabilitation professionals noted that workers need to learn new tasks, understand verbal or demonstrated instructions, remember operational sequences, and communicate with supervisors and peers during task performance.

“*They need to comprehend task steps, remember operational sequences, and communicate with supervisors, especially in their fast-paced or high-demand periods*,” a vocational rehabilitation professional explained (V4).

## 4 Discussion

This exploratory qualitative content analysis examined stakeholder-informed perspectives on employment-related factors, critical work tasks, and job demand domains in sewing work in China. The interview guide was designed to elicit stakeholder perspectives on sewing employment and key work tasks. However, the questions were generally framed at the level of work and employment conditions rather than as detailed prompts about workers' own individual workplace experiences. Therefore, the findings should be interpreted as stakeholder-informed accounts of sewing employment rather than as a comprehensive representation of workers' personal experiences. Given the exploratory nature of the study and the small, context-specific sample from one disability-inclusive sewing workplace, the findings should also be interpreted as context-specific insights rather than as generalizable findings. By combining dyadic interviews with workers and managers and individual interviews with vocational rehabilitation professionals, this study provides preliminary insights that may inform future vocational evaluation and SEWAbility system design.

To systematically categorize factors influencing employment outcomes (RQ 1), the PEOP framework was used to organize fourteen employment-related categories into three domains: Person, Environment, and Occupation. Performance was conceptualized as the outcome shaped by the interaction among these domains, referring to the ability to obtain and sustain sewing work. This classification highlights the multifaceted nature of employment, including personal characteristics, physical ability, workplace conditions, and sociocultural influences. The PEOP model supports interpretation of how person, environment, and occupation factors interact to shape employment performance in a real workplace context (31). For instance, sewing was once viewed as an accessible and popular career, yet employers today face recruitment challenges (24). Such shifts illustrate that employment-related factors are dynamic, influenced by social and economic changes. In addition to these employment-related categories, four critical work tasks were identified (RQ2): fabric side identification, manual stitching preparation, electric sewing machine operation, and instruction-based task execution. These tasks form the basis for understanding job demands (20). Vocational rehabilitation professionals further described these tasks as requiring gross and fine motor coordination, sensory ability, mental ability and communication (RQ 3).

The present findings show that semi-structured stakeholder interviews can provide useful insights into employment-related factors, critical work tasks, and broad job demand domains in sewing work. However, this qualitative interview-based approach was less suited to generating fine-grained task decomposition or measurable job-demand thresholds on its own. Participants identified important tasks, such as fabric side identification, manual stitching preparation, electric sewing machine operation, and instruction-based task execution, but they generally described these tasks at a broad functional level rather than as detailed sequences of observable work elements. Similarly, vocational rehabilitation professionals identified relevant physical, sensory, cognitive, and communication-related demands, but their descriptions did not provide precise quantitative indicators such as range of motion, movement speed, task rhythm, or repetitive motion thresholds. These limitations of interview-based stakeholder accounts indicate the need for complementary methods when detailed task decomposition and measurable job-demand indicators are required. Established approaches such as structured workplace observation and hierarchical task analysis may help capture task sequences and work elements more systematically (39, 40).

In addition, AI-enhanced video analysis may further support the quantification of observable physical and biomechanical demands, such as posture, movement range, repetitive motion, and task rhythm. However, the need for such complementary methods should not be interpreted as evidence that traditional job analysis as a whole is inadequate; rather, it indicates that interview-based qualitative accounts alone are insufficient when precise and task-specific job-demand indicators are required. This issue is particularly relevant in the Chinese vocational evaluation context, where available occupational information may not provide sufficiently detailed or locally specific job-demand data. Official documents such as China's National Occupational Classification (25) provide broad job duty descriptions, but they are typically too generic to reflect regional, factory-level, and task-specific differences. The U.S.-based O*NET database offers more detailed occupational data (41), but its applicability to the Chinese context is limited. For instance, some occupational categories or skill indicators (e.g., “electronic mail software”) may not align with local labor market practices (25, 42). Moreover, O*NET's sampling bias toward highly educated workers may inflate skill estimates (43). Consequently, when detailed local job-demand data are unavailable, vocational rehabilitation professionals may need to rely more heavily on clinical judgment, which can introduce subjectivity into vocational evaluation (44, 45).

The need for context-specific job-demand data also has implications for work-sample assessment and recruitment. Work-sample assessments are not job analysis methods themselves, but their validity depends on whether the selected tasks accurately represent real job requirements. Detailed job analysis can therefore support the design of more relevant work-sample assessments, which are valued for their face validity and client engagement compared with abstract ability measures (14, 15, 46). For example, the Pro3000 Modular Assessment System includes over 15 criterion-referenced modules mapped to more than 12,500 jobs in the DOT and O*NET databases (47). However, most validation studies of such tools are based in North America, limiting their applicability to regions with different labor market structures (48). In addition, standardized assessments may lag behind evolving job content, raising concerns about their current relevance. Work-sample tests should reflect real and dynamic job tasks supported by updated workplace data. Context-specific job-demand data may also support recruitment and person–job matching. In China, hiring decisions may rely on interviews, managerial judgment, or trial employment, which can be resource-intensive and may not systematically capture specific task demands (49, 50). While national policies emphasize aligning vocational evaluations with real job demands, current methods often remain too generic (5, 51). Therefore, more precise job-demand data are important for valid vocational evaluation, recruitment accuracy, and employment support for PWD. The present qualitative findings help specify what such data should include and how they may inform future SEWAbility use cases.

The findings inform SEWAbility development at three levels. The employment-related categories define the broader personal, environmental, and occupational context that cannot be captured through motion analysis alone. The four critical work tasks identify candidate scenarios for future work-sample or virtual reality-based assessment, while the four job demand domains help distinguish observable physical demands that may be quantified through video analysis from sensory, mental, and communication-related demands requiring complementary assessment. Broader psychological and contextual factors, such as motivation, family support, workplace atmosphere, cognitive fatigue, and stress regulation, also remain beyond the direct scope of pose estimation. Thus, the findings help define both the potential applications and the boundaries of SEWAbility. Within these defined boundaries, recent advances in AI and machine learning (ML) may provide complementary methodological support for improving the objectivity and precision of vocational evaluation, particularly when the target constructs are observable and measurable. ML techniques such as clustering, decision trees, and random forests have been applied to predict return-to-work outcomes by integrating physical, psychological, and environmental factors, and some studies have reported higher predictive performance than human assessments in specific tasks (52–54). For example, the Smart Work Injury Management (SWIM) system integrates variational autoencoders, principal component analysis, neural networks, and K-nearest neighbors to predict sick-leave duration, achieving 30% higher accuracy than human assessments in a validation study of 442 cases (55–57). Beyond prediction, ML may also support personalized treatment planning and real-time decision-making (58, 59). However, these examples should not be interpreted as evidence that AI can replace comprehensive vocational evaluation. The application of AI in healthcare and vocational rehabilitation remains constrained by limited labeled datasets, training-data bias, insufficient representativeness of development datasets, and reduced model robustness when systems are applied to new populations or real-world settings (60). Although transfer learning and self-supervised learning may reduce dependence on labeled data (61, 62), they do not remove the need for careful validation across different workers, disability profiles, task conditions, and workplace environments.

### 4.1 Limitations

This study has several limitations.

First, participants were recruited from a single sewing factory in China. Although the factory provided an information-rich case of disability-inclusive employment, its production scale, management practices, accessibility resources, institutional arrangements, and policy support may differ from those of other sewing workplaces. The transferability of the findings to other regions and organizational contexts is therefore limited. Future studies should include multiple factories with diverse workplace characteristics.

Second, the disability profiles represented in the worker–manager sample were limited. Only two participants reported holding registered disability certificates, and both had Grade 4 physical disabilities. Although participants were recruited as information-rich workplace stakeholders rather than to compare disability groups, the findings do not comprehensively represent workers with different disability types, severity levels, functional profiles, or support needs. Future research should recruit more diverse groups of workers with disabilities to examine how functional abilities and accommodation needs interact with sewing job demands.

Third, although the worker-manager sessions were initially planned as small group discussions, they are more accurately described as dyadic stakeholder interviews because each session involved one sewing worker and one manager. This format enabled the researchers to capture negotiated workplace perspectives on sewing employment, but it may also have shaped the data generated. In particular, the presence of managers may have limited workers' willingness to express divergent, critical, or personally sensitive views, especially given the hierarchical nature of workplace relationships. In addition, workers and managers were asked similar interview questions framed at a general level, which may have encouraged participants to discuss sewing employment from an observer perspective rather than eliciting workers' personal workplace experiences in depth. The limited presence of contradictory or alternative worker perspectives in the findings should therefore be considered when interpreting the results. Future research should consider conducting separate worker-only focus groups or individual interviews using more experience-based prompts to better capture workers' own experiences, challenges, and support needs.

Fourth, vocational rehabilitation professionals were shown workplace videos as reference materials during the individual interviews. Although this approach helped stimulate discussion and reflects aspects of vocational rehabilitation practice when direct workplace assessment is not feasible, the experts' responses may have been influenced by the scope, quality, and representativeness of the selected videos. Because video materials primarily display observable work behaviors, their use may have contributed to greater emphasis on physical, biomechanical, and sensory demands than on less visible psychological, cognitive, interpersonal, or emotional demands. Although mental ability and communication emerged as a job-demand domain, factors such as sustained attention, cognitive fatigue, tolerance of repetitive work, stress and emotional regulation, and adaptation to production pressure were not explored in sufficient depth. This omission is important because high cognitive workload can impair performance in manufacturing assembly tasks, while physical and psychological job demands have been associated with fatigue, particularly mental fatigue, among industrial workers whose work involves repetitive and concentration-intensive activities (63, 64). Future research should examine these demands more explicitly, particularly among workers with intellectual or psychosocial disabilities.

Fifth, although real-time participant confirmation was conducted at the end of each interview, formal transcript-level or findings-level member checking was not undertaken. Participants were not invited to review full transcripts, coding categories, or preliminary interpretations after data analysis. This may have limited opportunities for participants to verify, refine, or challenge the researchers' interpretation of the data. Future studies should consider incorporating more formal member checking procedures, particularly when exploring workers' personal workplace experiences or potentially sensitive workplace issues.

Finally, AI-enhanced video-based job analysis cannot replace comprehensive vocational evaluation. The planned SEWAbility system is limited to quantifying observable and video-extractable physical job demands, whereas factors such as motivation, learning ability, family support, workplace atmosphere, communication, and psychological adjustment require other assessment methods. Accordingly, SEWAbility should be used as a complementary decision-support tool alongside professional judgment, worker interviews, contextual assessment, and individualized vocational rehabilitation planning.

### 4.2 Future research directions

Building on the exploratory findings of this qualitative study, future research will advance the development and validation of the SEWAbility system as an AI-enhanced, video-based decision-support framework for vocational evaluation (23). Its initial technical focus will be the quantification of observable and video-extractable physical job demands that are difficult to measure precisely through interviews alone.

The next stage of research will focus on translating the present findings into system design. The four critical work tasks identified in this study (fabric side identification, manual stitching preparation, electric sewing machine operation, and instruction-based task execution) will inform the selection of representative sewing scenarios for future work-sample or virtual reality-based vocational evaluation. The physical components of the job demand domains will guide the extraction of video-based motion features, such as posture, range of motion, movement speed, task rhythm, repetitive motion frequency, and joint movement patterns. At the same time, the employment-related categories and non-physical job demand domains will inform the design of complementary assessment modules that capture contextual, cognitive, motivational, interpersonal, and psychosocial factors beyond the scope of pose estimation.

Future SEWAbility development will include two core modules: a job analysis module and a work ability assessment module. The job analysis module will use workplace videos and pre-trained human pose estimation models, such as ViTPose and OpenPose (65, 66), to extract biomechanical indicators, including joint angles, postures, and motion trajectories, thereby supporting the quantification of physical job demands. The work ability assessment module will then compare an individual's observed task performance against these job-demand benchmarks, potentially within a virtual reality or simulated work-sample environment, to generate quantitative indicators of task fit and identify areas requiring support, training, or accommodation.

The subsequent validation stage should adopt a multi-level strategy to examine the measurement quality, usability, and transferability of SEWAbility. Criterion-related validity should be examined by comparing SEWAbility outputs with concurrent external criteria, including expert ratings, observed workplace performance, work-sample outcomes, and trial-employment results. Future studies should also examine whether the system can distinguish between workers who are more or less suitable for specific sewing tasks, as well as between experienced workers and new entrants. Test–retest reliability should be evaluated to determine whether the system produces stable results when the same worker performs comparable tasks under similar conditions. In addition, user testing should be conducted with vocational rehabilitation professionals, employers, and workers with and without disabilities. Usability and acceptance may be assessed using standardized tools such as the System Usability Scale (67) and the Technology Acceptance Model (68), supplemented by qualitative feedback on interpretability, workflow integration, perceived usefulness, and ethical concerns. Validation should include diverse participants with different disability types, severity levels, functional profiles, and accommodation needs, and should test system robustness across different camera angles, lighting conditions, clothing, postures, task conditions, and factory environments. Although sewing work was selected as the initial pilot occupation, future research should examine whether the SEWAbility framework can be adapted to other labor-intensive occupations through occupation-specific job analysis, task selection, feature extraction, and validation. Collectively, these studies will determine whether SEWAbility can provide reliable, valid, usable, and transferable support for vocational evaluation and person–job matching across sewing and other labor-intensive occupations.

## 5 Conclusions

This study employed a two-tiered exploratory qualitative design, including three dyadic stakeholder interviews and four individual interviews, to examine employment-related factors, critical work tasks, and job demand domains in sewing work. The findings provide context-specific, stakeholder-informed insights into disability-inclusive sewing employment in China. They also suggest that semi-structured stakeholder interviews can identify broad work-related categories, but should be complemented by structured observation, task analysis, and AI-enhanced video analysis when detailed task decomposition and measurable physical job-demand indicators are required. These insights may inform the development of more structured, context-sensitive, and evidence-informed approaches to vocational evaluation and person–job matching. Future AI-enhanced tools, such as SEWAbility, should be positioned as complementary decision-support approaches for quantifying observable physical job demands, while remaining integrated with professional judgment, worker interviews, contextual assessment, and individualized rehabilitation planning.

## Data Availability

The original contributions presented in the study are included in the article, further inquiries can be directed to the corresponding authors.
